# Streamlining the Non-operative Management of Adhesive Small Bowel Obstruction: A Closed-Loop Audit Evaluating the Introduction of a Standardised Gastrografin Protocol in a District General Hospital

**DOI:** 10.7759/cureus.73327

**Published:** 2024-11-09

**Authors:** Saif Rehman, Yousif Baho, Apoorva Thakur, Eriberto Farinella

**Affiliations:** 1 General Surgery, Lister Hospital, East and North Hertfordshire NHS Trust, Stevenage, GBR

**Keywords:** adhesive small bowel obstruction, asbo, gastrografin, gastrografin protocol, sbo, small bowel obstruction

## Abstract

Introduction

Adhesive small bowel obstruction (ASBO) is a common cause of admissions to general surgery services. Water-soluble contrast (WSC), such as Gastrografin® (GGF), can be utilised in the conservative management of patients with ASBO, with predictive and possible therapeutic value. We audited the non-operative management of ASBO in the general surgery department of East and North Hertfordshire NHS Trust, a district general hospital. Following the introduction of a hospital-wide protocol standardising the use of GGF in patients with ASBO, a re-audit was conducted to assess its impact.

Methods

A retrospective audit of ASBO patients who received GGF was conducted in two cycles: before and after the implementation of the protocol. Data were collected between February and June 2022 for group 1 (pre-protocol) and between August and November 2023 for group 2 (post-protocol).

Results

Forty-eight patients were included in the analysis across both groups: 31 in group 1 (pre-protocol) and 17 in group 2 (post-protocol). The success rate of this non-operative trial with GGF increased from 77% (24/31) in group 1 to 88% (15/17) in group 2. The median duration of conservative management was two days in both groups. The median time from CT scan to GGF administration decreased from 24 hours in group 1 to 19 hours 22 minutes in group 2, and the interval between GGF administration and first abdominal radiograph decreased from 9 hours in group 1 to 6 hours 25 minutes in group 2.

Conclusion

Our closed-loop audit suggests that by streamlining the non-operative management pathway of adhesive small bowel obstruction, our standardised protocol is safe and possibly improves efficiency within a district general hospital setting.

## Introduction

Small bowel obstruction (SBO) is a frequent general surgery presentation, accounting for up to 20% of surgical admissions [1]. Adhesions are the most frequent aetiology, responsible for around 60% of post-operative SBO cases [2]. Adhesions are presumed to be the cause of small bowel obstruction where there is a history of previous abdominal surgery and indicative findings on CT imaging, such as the absence of other causes, i.e. malignancy or hernias [3].

Non-operative management of adhesive small bowel obstruction (ASBO) can be safely trialled in most cases with a success rate of 60-80% [4,5], except when there are features of peritonitis, bowel strangulation, or ischaemia, requiring emergency surgery [6]. This typically involves decompression with a nasogastric (NG) tube or long intestinal tube, nil by mouth, fluid therapy and electrolyte correction [6,7]. Additionally, water-soluble contrast (WSC) such as Gastrografin® (GGF) can be safely administered providing predictive and likely therapeutic value. The presence of WSC in the colon strongly predicts the resolution of SBO with 96% sensitivity and 98% specificity, whilst administration reduced the need for surgery by 9% [8].

The 2018 Bologna guidelines for the diagnosis and management of ASBO recommend a trial of non-operative management in all ASBO patients unless contraindicated, with a maximum duration of 72 hours [6]. There is limited evidence on the optimal duration; however, it is generally considered that the increased risk of mortality and morbidity with delayed surgery outweighs the potential benefits of extending medical management beyond this period [9]. The authors therefore recommend surgical intervention, preferably laparoscopic, if WSC is not present in the colon after 24-36 hours. In the UK, the National Audit of Small Bowel Obstruction (NASBO) report supports these recommendations, specifically the use of WSC for both predictive and therapeutic purposes, and reinforces the 72-hour limit for conservative management [10].

In line with these guidelines, we audited the non-operative management of ASBO patients in the general surgery department of a district general hospital. Following this, we introduced a hospital-wide protocol to standardise the use of Gastrografin® in ASBO patients not requiring emergency surgery. We subsequently re-audited after protocol implementation to assess its impact on several key metrics in the management pathway, aiming for a reduction in time to GGF administration, the requirement of surgical intervention, conservative management duration, as well as ensuring appropriate timing of post-contrast abdominal radiographs.

## Materials and methods

We performed a retrospective audit of patients who received Gastrografin® (GGF) in a single centre at East and North Hertfordshire NHS Trust. The audit was carried out in two cycles, before (group 1) and after (group 2) GGF protocol implementation, between February and June 2022 and August and November 2023, respectively. Patients were identified by screening a list of all abdominal radiographs performed within the time periods for “Gastrografin” or other related search terms. Additional cases found incidentally were also included.

We included patients admitted to general surgery with a diagnosis of adhesive small bowel obstruction (ASBO) and who received Gastrografin®. The diagnosis was radiologically determined by CT findings, in combination with clinical presentation and surgical history. We excluded patients who were younger than 18 or pregnant. Patients who had a diagnosis of large bowel obstruction, ileus, or small bowel obstruction of non-adhesive aetiology (herniation, stricture, or compression by mass) were also excluded. Finally, we excluded patients who were admitted and partly managed under medical specialities.

Relevant data was then collected from electronic patient records. As the hospital transitioned from paper to electronic prescribing in 2022, it was not possible to determine the time of GGF administration in earlier cases, with no documentation of administration in the clinical notes. These were therefore excluded from analysis specifically relating to time intervals to and from GGF administration.

**Figure 1:**
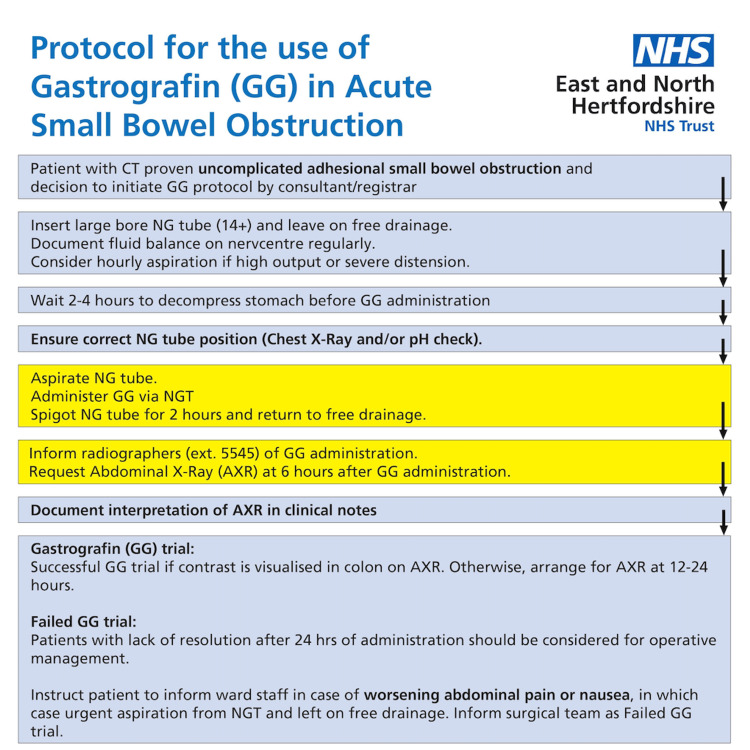
Protocol for the use of Gastrografin® in adhesive small bowel obstruction Protocol created by the authors and launched on general surgery wards and the surgical assessment unit at the end of July 2023

The results of the initial cycle were presented at a local general surgery departmental meeting. Following consultation with general surgery and radiology department stakeholders, a Gastrografin® protocol was launched and displayed on surgical wards at the end of July 2023 (Figure 1). Patients were then identified retrospectively for the second cycle, with results analysed and compared to the first audit cycle.

## Results

A total of 48 patients with adhesive small bowel obstruction who received Gastrografin® were included across both cycles; 31 in the pre-protocol cycle (group 1) and 17 in the post-protocol cycle (group 2).

The results of the included patients are summarised in Table 1.

**Table 1:** Included ASBO patients who received GGF Comparison of patients included in the first cycle prior to protocol implementation (Group 1) and the second cycle after protocol implementation (Group 2). Age and time metrics are reported as median values with the range of minimum and maximum values in brackets. Absolute values are reported as the number of patients, with the proportion out of the group included as a percentage in brackets. ASBO = Adhesive Small Bowel Obstruction, GGF = Gastrografin®, ED = Emergency Department, NG = Nasogastric, AXR = Abdominal X-Ray

Patient demographics and metrics	Pre-protocol cycle (Group 1), n=31	Post-protocol cycle (Group 2), n=17
Age (years)	64 (22-93)	73 (38-99)
Gender, males	9 (29%)	9 (53%)
Gender, females	22 (71%)	8 (47%)
Successful resolution	24 (77%)	15 (88%)
Failed trial requiring surgery	7 (23%)	2 (12%)
Non-operative management duration (days)	2 (1-5)	2 (0.5-5.5)
Time to CT from ED arrival (hours and minutes)	5hr 49 min (2 hrs 19 min – 133 hrs 51 min)	6 hrs 4 min (0 hr 50 min – 10 hrs 20 min)
Time from CT scan to GGF administration (hours and minutes)	24 hrs (3 hrs – 96 hrs)	19 hrs 22 min (4 hrs 23 min – 86 hrs 55 min)
Correct NG tube position confirmed radiographically	24 (77%)	11 (65%)
Time from GGF administration to first AXR (hours and minutes)	9 hrs (3 hrs 30 min – 16 hrs)	6 hrs 25 min (4hrs 4 min – 25 hrs 32 min)
Second AXRs conducted	10 (32%)	10 (59%)
Time from first AXR to second AXR (hours and minutes)	7 hrs 30 min (4 hrs – 40 hrs)	6 hrs 40 min (2 hrs 9 min – 33 hrs 15 min)

The success rate of bowel obstruction resolution following the Gastrografin® trial was 77% (24/31) in group 1 and 88% (15/17) in group 2. In group 1, all 7 patients (23%) who failed the GGF trial underwent laparoscopic adhesiolysis surgery and in group 2, both patients (12%) who failed the GGF trial underwent laparoscopic adhesiolysis. There were no intra-operative findings of intestinal ischaemia.

The median total length of non-operative management was two days for both groups. This was defined as the time in days between NG tube insertion and removal, either due to a successful GGF trial or prior to operative management in failed trials.

We were unable to determine the Gastrografin® administration time in the 7 earliest cases included in group 1, so only 24 patients (77%) were included when reporting the median time from CT scan to GGF administration or the time from GGF administration to first abdominal radiograph. The median time from CT scan to GGF administration was 24 hours in the pre-protocol cycle (group 1) and 19 hours 22 minutes in the post-protocol cycle (group 2). The median time from GGF administration to the first abdominal radiograph was 9 hours for group 1 and 6 hours 25 minutes for group 2.

## Discussion

Our closed-loop audit aimed to assess the non-operative management of adhesive small bowel obstruction (ASBO) using Gastrografin® (GGF) and evaluate the impact of a standardised protocol on key metrics in a district general hospital. The protocol was designed to streamline the management pathway, ensuring safe and efficient decision-making to improve patient outcomes.

The results from both audit cycles demonstrate adherence to international guidelines and a possible positive impact of the protocol, although the relatively small sample size limited statistical power for comparison between groups. The success rate of non-operative management in ASBO patients increased from 77% (24/31) in the pre-protocol group (group 1) to 88% (15/17) in the post-protocol group (group 2). This is consistent with the literature reporting a success rate of 60-80% for conservative management [4,5].

Protocol implementation was potentially responsible for the 4.5-hour reduction in the median time from CT scan to GGF administration, from 24 hours to 19 hours 22 minutes. This helped maintain a median conservative management duration of 2 days in both groups, well within the 72-hour limit proposed by the Bologna guidelines and the National Audit of Small Bowel Obstruction (NASBO) report [6,10]. Additionally, there is evidence that early administration of GGF may reduce complications and one-year mortality rate [11]. In terms of cost, a Dutch study found an overall cost of €2277 for non-operative treatment (mean four-day hospital stay) [12]. Given the high burden of ASBO on general surgery services, reducing the length of hospital stay could yield significant overall cost savings [1].

The median time to first abdominal radiograph (AXR) decreased from 9 hours in group 1 to 6 hours 25 minutes in group 2, aligning with the 6-hour target recommended by our protocol and existing evidence of high sensitivity, specificity, negative predictive value (NPV), and positive pressure ventilation (PPV) of early AXRs [8]. However, we observed a significant increase in the proportion of second AXRs in group 2, from 10/31 (32%) to 10/17 (59%). This was largely due to simultaneous requests for both the first and second AXRs, which were not in line with the protocol. We re-emphasized to the department that second AXRs should only be requested if contrast is not visible in the colon on the first AXR, and potential future audits could assess adherence to this. Alternatively, the radiology department could consider only performing the second AXR when clinically confirmed as necessary.

Although not the focus of our audit, the median time to CT from arrival in ED was 5 hours 49 minutes in group 1 and 6 hours 4 minutes in group 2. While this is dependent on patient flow within the emergency department, the NASBO report recommends early use of CT scanning, in preference to abdominal radiographs, due to diagnostic and prognostic reasons [10]. All our included patients received CT scans for the initial diagnosis.

A few studies have evaluated the impact and safety of standardised non-operative management protocols for patients with ASBO [13,14]. Long et al. compared outcomes between a hospital using a protocol and a sister hospital without one, demonstrating lower surgery rates and shorter duration of hospitalisation in the protocol-driven setting [13]. Maienza et al. assessed the safety and effectiveness of their protocol in a cohort of 661 patients over 14 years, reporting a 64% success rate with only a 0.9% risk of late small bowel resection for intestinal ischaemia [14]. While these findings are consistent with our own audit, our study uniquely focuses on specific metrics related to the management pathway, such as time to Gastrografin® administration, time to abdominal radiographs, and the rate of second AXR requests, providing further insights into operational efficiency.

Limitations

Several limitations should be considered. As previously mentioned, the small sample size limited our ability to detect statistically significant differences between the pre and post-protocol groups though the results suggest general adherence to international guidelines. Additionally, the data was collected retrospectively in a non-blinded, non-randomised manner, and we may have missed some ASBO cases by relying on GGF-related search terms. Moving forward, a prospective study with larger sample sizes could address these limitations. Despite these limitations, we feel our audit still demonstrates a safe, effective and easy-to-follow protocol for the non-operative management of ASBO that can be applied in a smaller district general hospital setting.

## Conclusions

The findings from this closed-loop audit suggest that our standardised protocol for the non-operative management of adhesive small bowel obstruction is safe in a district general hospital setting and possibly improves efficiency in key metrics, including a reduction in time to Gastrografin® administration and time to initial abdominal radiograph. Given this, the protocol was recommended for continued use in the department. Whilst our results are promising, larger prospective studies are needed to confirm these findings and further investigate long-term outcomes such as readmission rates.
